# Supplementary material for: Regulation of PP2A, PP4, and PP6 holoenzyme assembly by carboxyl-terminal methylation
Source: Sci Rep. 2021 Nov 29;11:23031. doi: 10.1038/s41598-021-02456-z (PMC8630191; doi:10.1038/s41598-021-02456-z)
Supplement: Supplementary file 1 — Supplementary Figure 1. [file 41598_2021_2456_MOESM1_ESM.pdf]

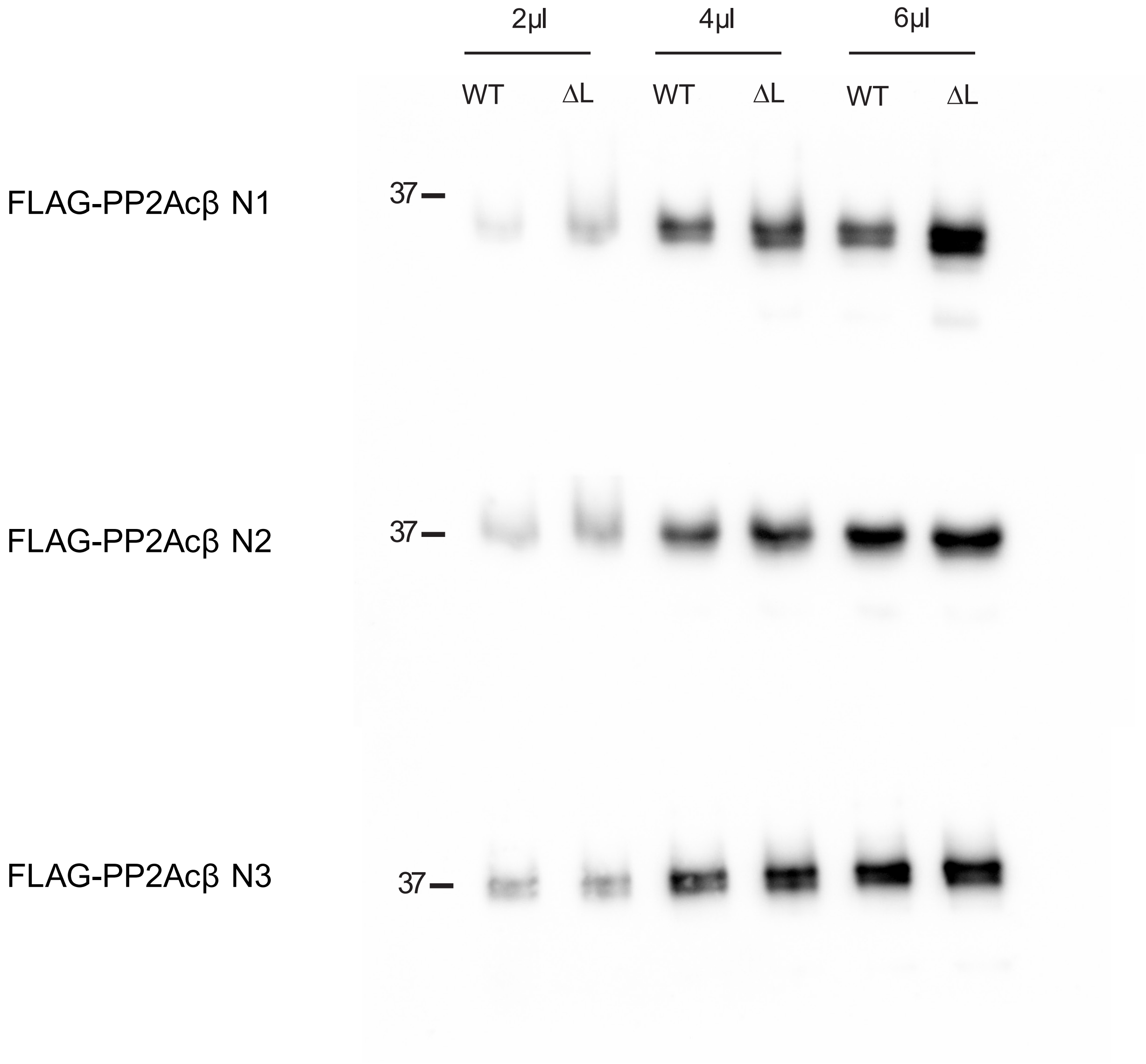

Supp. Figure 1. Purification of wild-type or ΔL mutant catalytic subunits of PP2A. Western blots of affinity purified FLAG-PP2Acβ wild-type and ΔL mutant.
