# Supplementary material for: Regulation of PP2A, PP4, and PP6 holoenzyme assembly by carboxyl-terminal methylation
Source: Sci Rep. 2021 Nov 29;11:23031. doi: 10.1038/s41598-021-02456-z (PMC8630191; doi:10.1038/s41598-021-02456-z)
Supplement: Supplementary file 2 — Supplementary Figure 2. [file 41598_2021_2456_MOESM2_ESM.pdf]

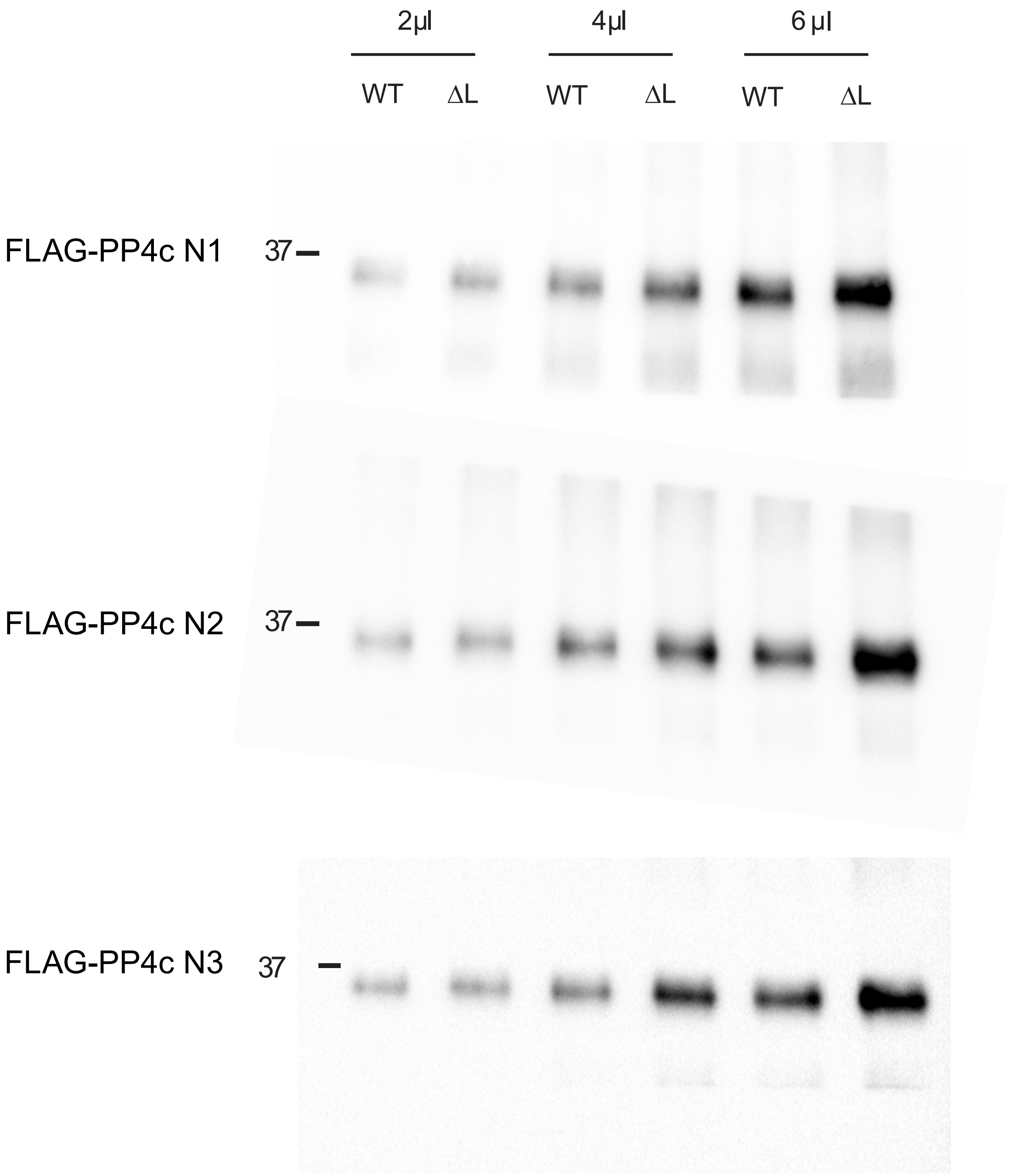


**Supp. Figure 2**. Purification of wild-type or ΔL mutant catalytic subunits of PP4. Western blots of affinity purified FLAG-PP4c wild-type and ΔL mutant.
