# Supplementary material for: Regulation of PP2A, PP4, and PP6 holoenzyme assembly by carboxyl-terminal methylation
Source: Sci Rep. 2021 Nov 29;11:23031. doi: 10.1038/s41598-021-02456-z (PMC8630191; doi:10.1038/s41598-021-02456-z)

293T

Total PP2Acα/β

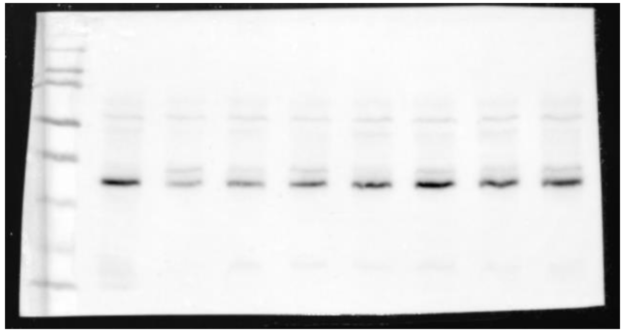

*

WT HM WT HM WT HM WT HM

Ponceau

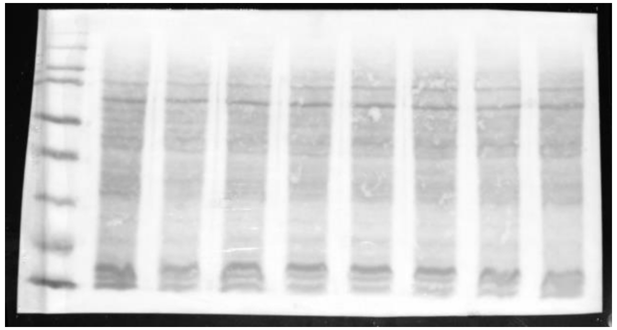

WT HM WT HM WT HM WT HM

Methylated PP2Acα/β

250
150
100
75
50
37
25
20

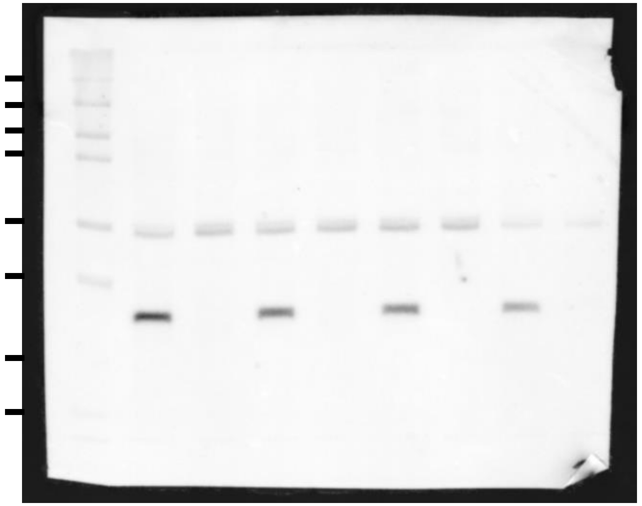

*

WT HM WT HM WT HM WT HM

Ponceau

250
150
100
75
50
37
25
20

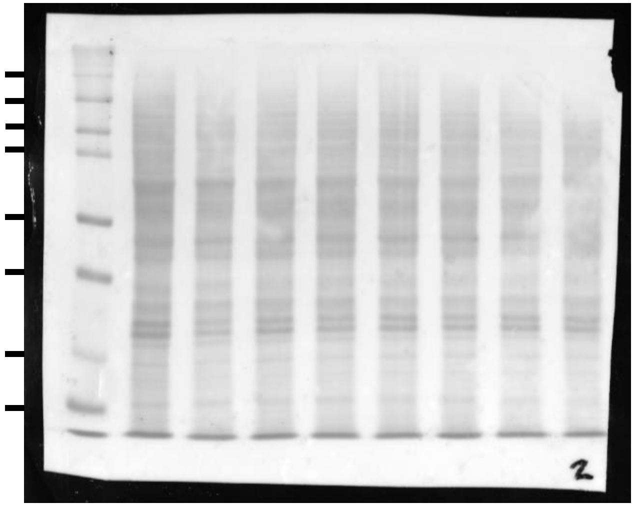

WT HM WT HM WT HM WT HM

* non-specific band

293T

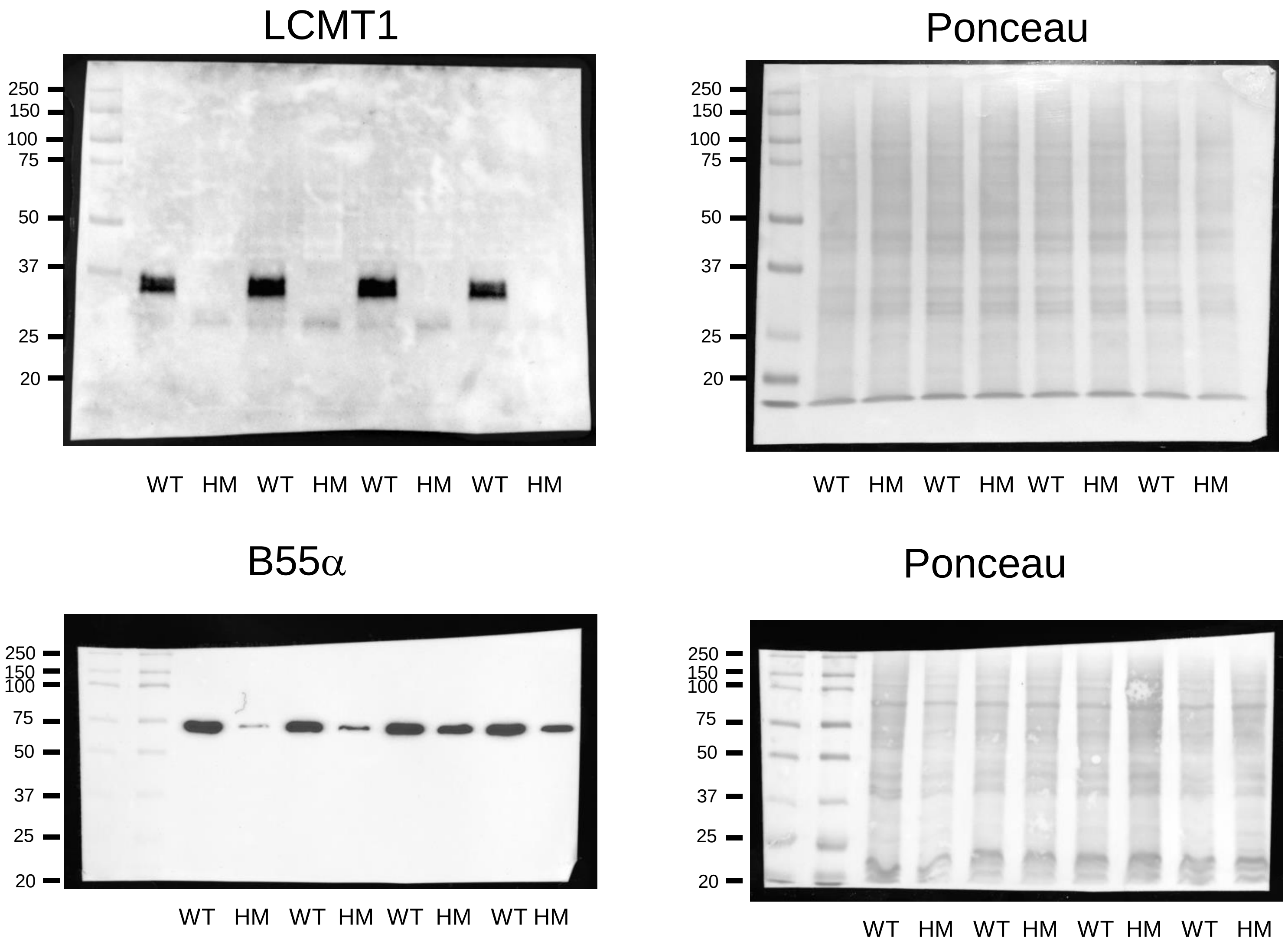
LCMT1
Ponceau
B55α
Ponceau
250
150
100
75
50
37
25
20
WT HM WT HM WT HM WT HM

HeLa

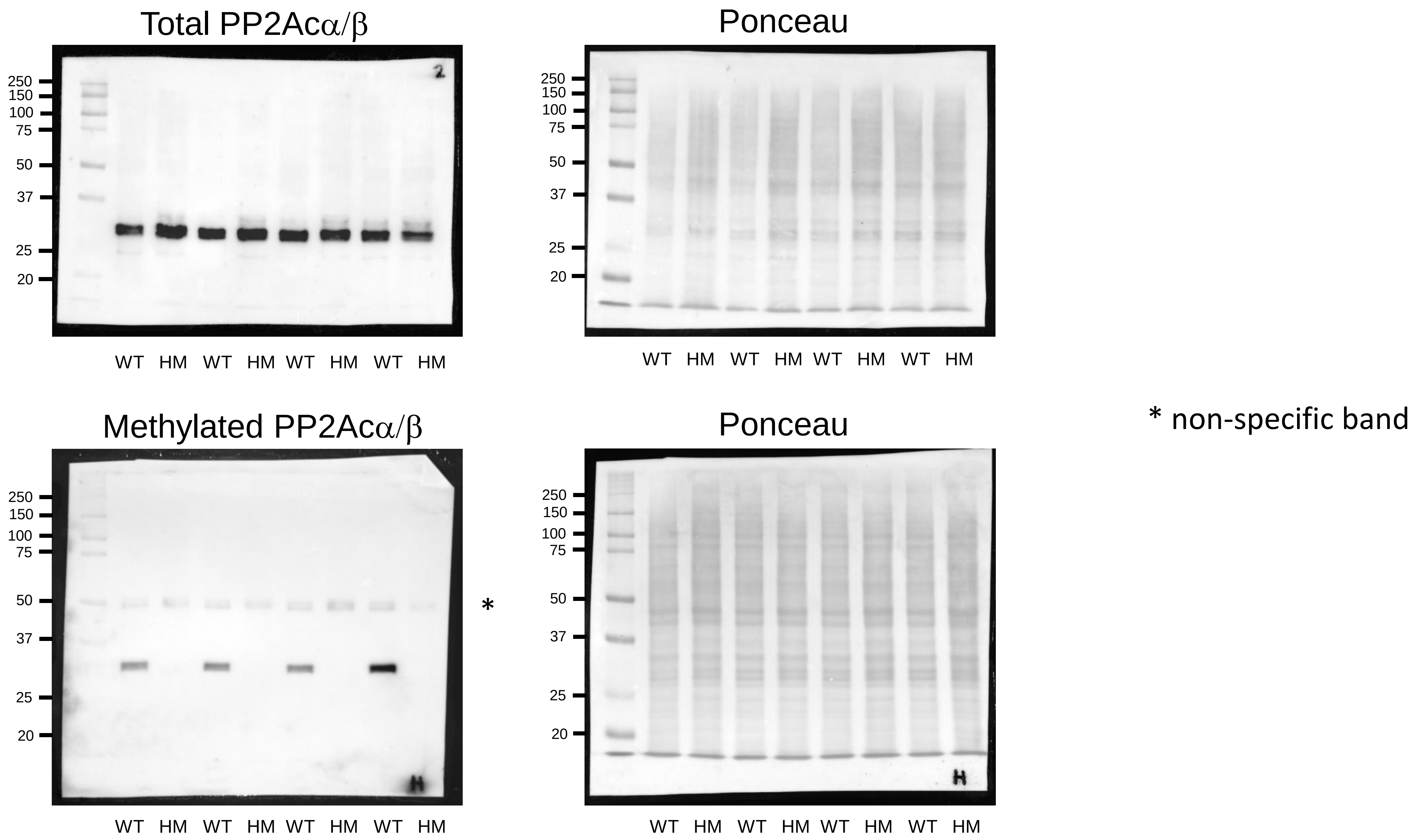
Total PP2Acα/β
Ponceau
250
150
100
75
50
37
25
20
2
WT HM WT HM WT HM WT HM
Methylated PP2Acα/β
Ponceau
*
H
* non-specific band

HeLa

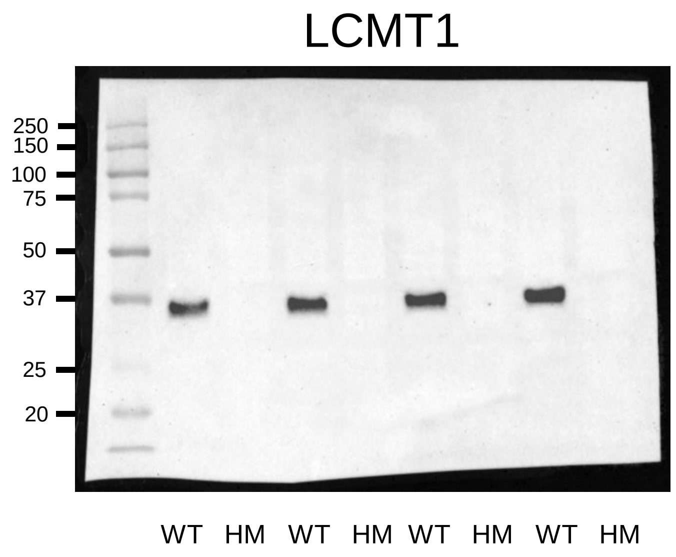
LCMT1
250
150
100
75
50
37
25
20
WT HM WT HM WT HM WT HM

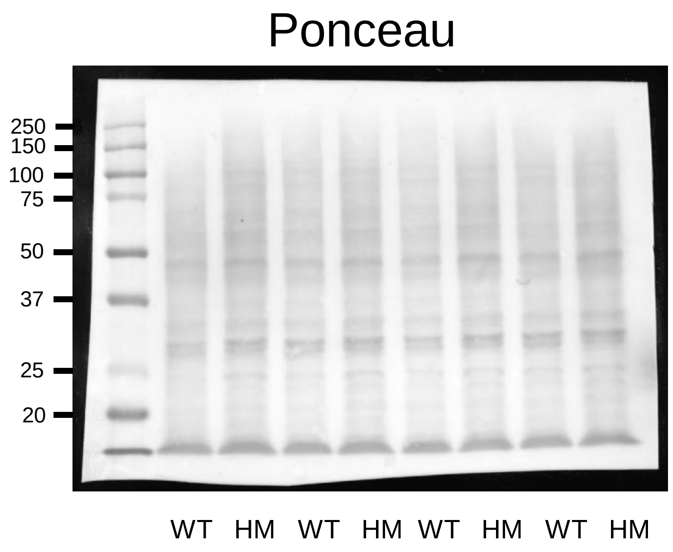
Ponceau
250
150
100
75
50
37
25
20
WT HM WT HM WT HM WT HM

Rpe1

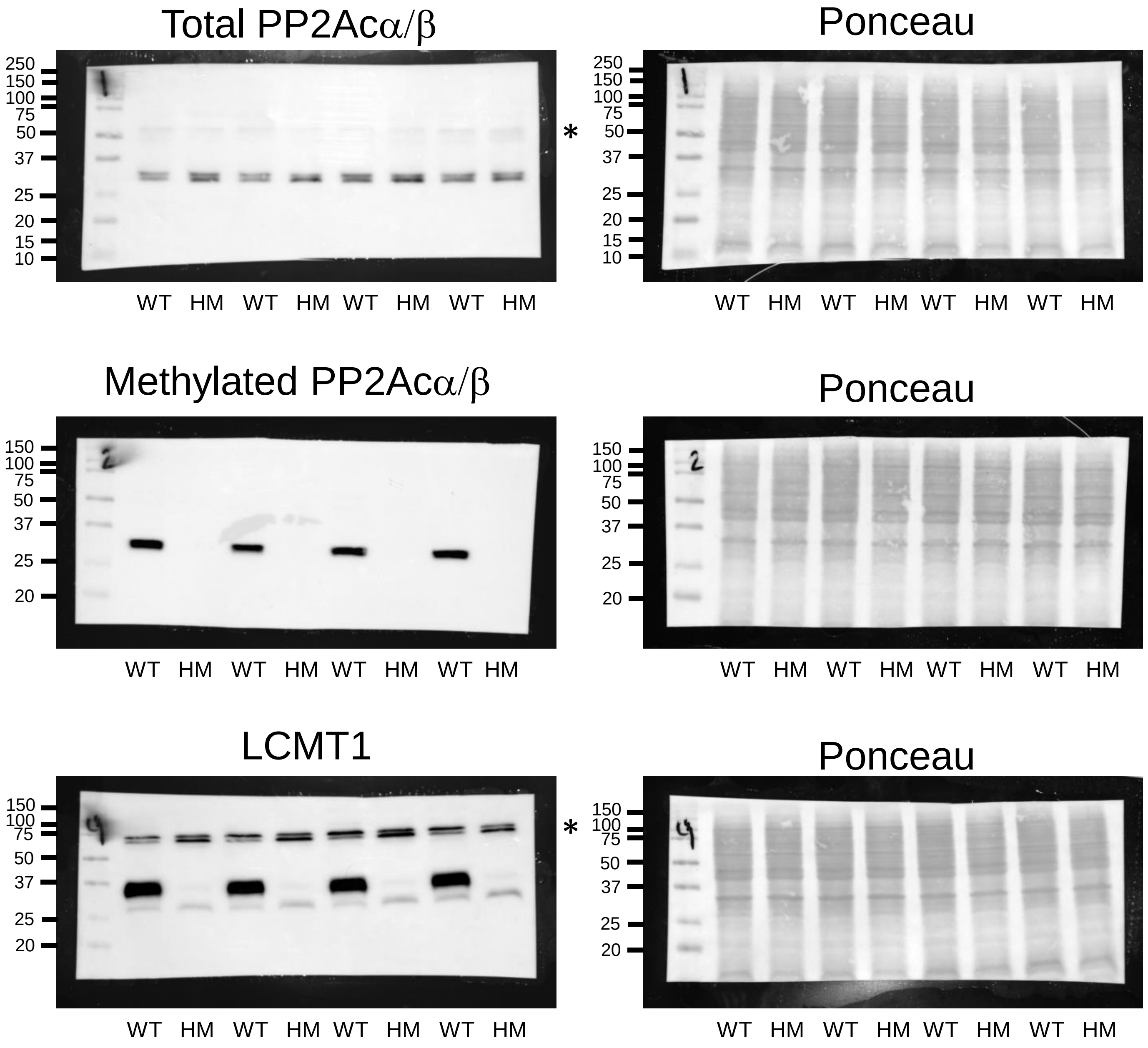

* non-specific band

Supplemental Figure 1: FLAG-PP2Acβ blots

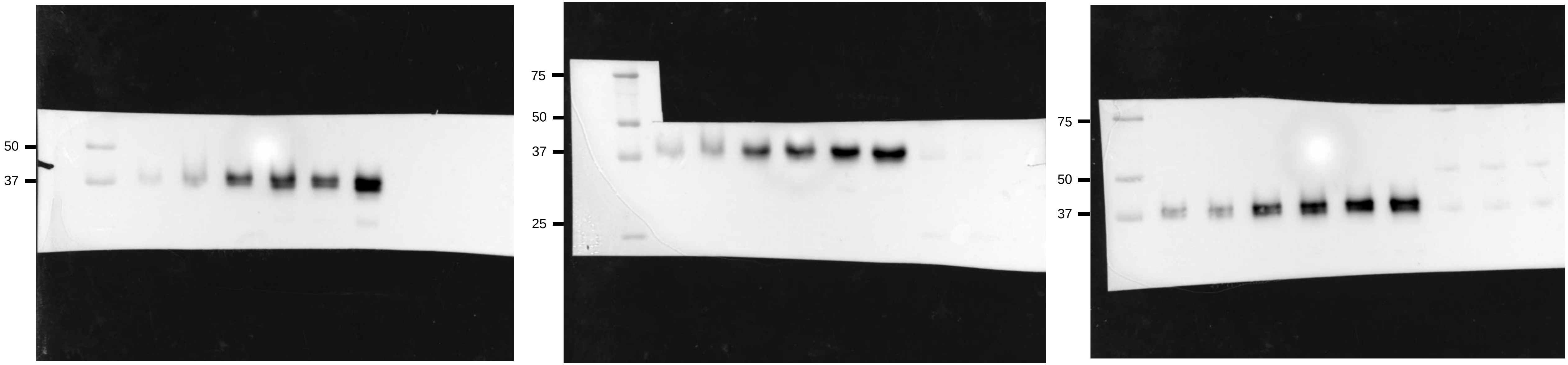

Supplemental Figure 1: FLAG-PP4c blots

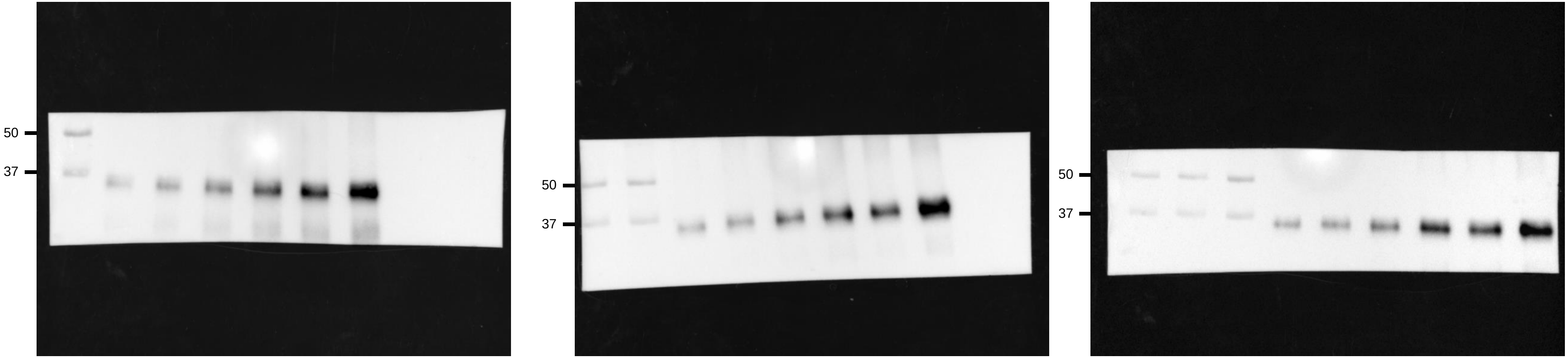

Supplemental Figure 1: FLAG-PP6c blots

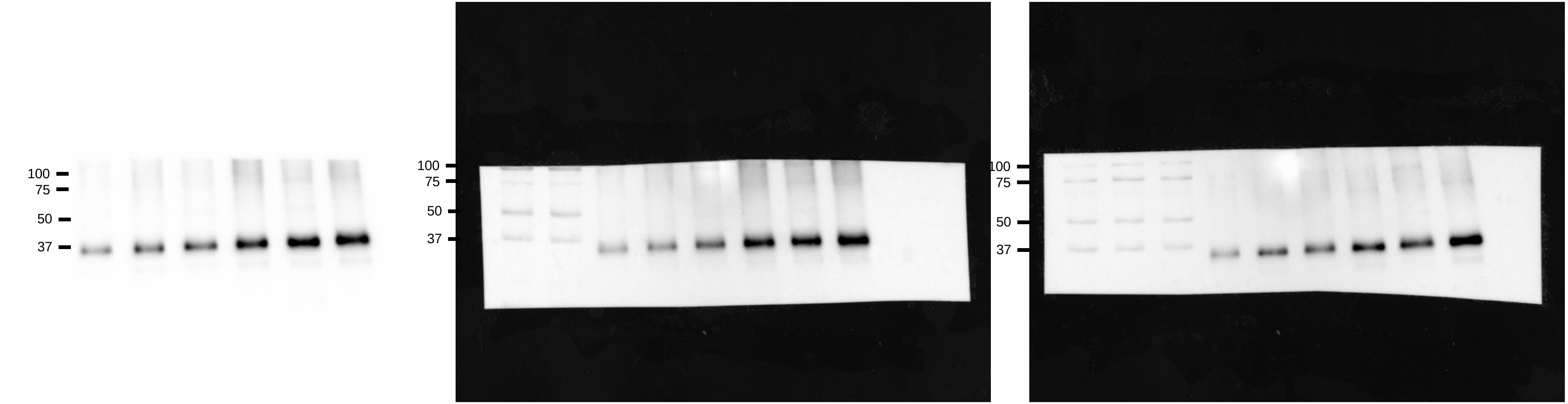

Supplement: Supplementary file 11 — Supplementary Information 1. [file 41598_2021_2456_MOESM11_ESM.pdf]
